# Male Goal-Tracker and Sign-Tracker Rats Do Not Differ in Neuroendocrine or Behavioral Measures of Stress Reactivity

**DOI:** 10.1523/ENEURO.0384-20.2021

**Published:** 2021-05-06

**Authors:** Sofia A. Lopez, Eman Mubarak, Charlotte Yang, Aram Parsegian, Marin Klumpner, Paolo Campus, Shelly B. Flagel

**Affiliations:** 1 Neuroscience Graduate Program; 2 Undergraduate Program in Neuroscience; 3 Michigan Neuroscience Institute; 4 Psychiatry Department, University of Michigan, Ann Arbor, MI 48109

**Keywords:** corticosterone, glucocorticoid receptors, incentive salience, stress reactivity

## Abstract

Environmental cues attain the ability to guide behavior via learned associations. As predictors, cues can elicit adaptive behavior and lead to valuable resources (e.g., food). For some individuals, however, cues are transformed into incentive stimuli and elicit motivational states that can be maladaptive. The goal-tracker (GT)/sign-tracker (ST) animal model captures individual differences in cue-motivated behaviors, with reward-associated cues serving as predictors of reward for both phenotypes but becoming incentive stimuli to a greater degree for STs. While these distinct phenotypes are characterized based on Pavlovian conditioned approach (PavCA) behavior, they exhibit differences on a number of behaviors relevant to psychopathology. To further characterize the neurobehavioral endophenotype associated with individual differences in cue-reward learning, neuroendocrine and behavioral profiles associated with stress and anxiety were investigated in male GT, ST, and intermediate responder (IR) rats. It was revealed that baseline corticosterone (CORT) increases with Pavlovian learning, but to the same degree, regardless of phenotype. No significant differences in behavior were observed between GTs and STs during an elevated plus maze (EPM) or open field test (OFT), nor were there differences in CORT response to the OFT or physiological restraint. Upon examination of central markers associated with stress reactivity, we found that STs have greater glucocorticoid receptor (GR) mRNA expression in the ventral hippocampus, with no phenotypic differences in the dorsal hippocampus or prelimbic cortex (PrL). These findings demonstrate that GTs and STs do not differ on stress-related and anxiety-related behaviors, and suggest that differences in neuroendocrine measures between these phenotypes can be attributed to distinct cue-reward learning styles.

## Significance Statement

While the goal-tracker (GT)/sign-tracker (ST) animal model derives from individual differences in Pavlovian conditioned approach (PavCA) behavior, other traits, including some of relevance to addiction and post-traumatic stress disorder (PTSD), have been shown to co-exist with the propensity to sign-track. The extent to which this model encompasses differences in aversive arousal and associated neuroendocrine measures, however, remains largely unexplored. Here, we show that behavioral and corticosterone (CORT) response to stress-related paradigms do not differ between GTs and STs. However, glucocorticoid receptor (GR) expression in the ventral hippocampus does differ between phenotypes, suggesting that this central marker that is typically associated with stress responsivity, may, in fact, play an important role in appetitive motivation.

## Introduction

Through learned associations, environmental cues become predictors of biologically relevant stimuli. In turn, such cues elicit an adaptive response, facilitating behavior toward valuable resources. For some individuals, however, cues elicit complex emotional responses and can prompt maladaptive behavior. For example, upon exposure to drug-associated cues, individuals with addiction report drug-craving and, consequently, often relapse (Ehrman et al., 1992). Similarly, when exposed to trauma-related stimuli, individuals with post-traumatic stress disorder (PTSD) report hyperarousal and anxiety (Shin et al., 2004). Cues attain the ability to elicit extreme emotional states and aberrant behavior when they are attributed with excessive incentive motivational value, or incentive salience (Robinson and Berridge, 1993). The propensity to attribute incentive salience to environmental cues, thereby, may reflect a vulnerability trait for cue-motivated psychopathologies, like addiction and PTSD (Flagel et al., 2010; Morrow et al., 2011).

Individual variation in the propensity to attribute incentive salience to reward cues can be captured using a Pavlovian conditioned approach (PavCA) paradigm, consisting of a lever-cue paired with delivery of a food-reward (Robinson and Flagel, 2009). Upon lever-cue presentation, goal-trackers (GTs) direct their behavior toward the location of reward delivery, whereas sign-trackers (STs) approach the cue itself. For both GTs and STs the cue attains predictive value, but for STs the cue also attains incentive value and is transformed into a “motivational magnet” (Berridge et al., 2009). Intermediate responders (IRs) vacillate between goal-directed and cue-directed behavior, without preference for either cue-learning strategy. GTs and STs differ on a number of traits of relevance to psychopathology. Relative to GTs, STs are more impulsive (Lovic et al., 2011), exhibit an exaggerated fear response to aversive stimuli (Morrow et al., 2011, 2015), show poor attentional control (Paolone et al., 2013), and have a greater propensity for reinstatement of drug-seeking behavior (Flagel et al., 2010; Saunders and Robinson, 2010, 2011; Saunders et al., 2013; Yager and Robinson, 2013; also see Kawa et al., 2016). These behavioral phenotypes are subserved by distinct neural mechanisms (Flagel et al., 2011b; Pitchers et al., 2017; Campus et al., 2019). While GTs seem to rely on “top-down” cortical control, STs are presumed to be driven by subcortical “bottom-up” circuitry (Flagel and Robinson, 2017; Kuhn et al., 2018; Sarter and Phillips, 2018). Thus, the GT/ST model captures a neurobehavioral endophenotype reflective of more than individual differences in cue-reward learning.

Most of the research surrounding the GT/ST model has focused on appetitive motivation, with only a few studies investigating indices of aversive arousal (Morrow et al., 2011; Harb and Almeida, 2014; Vanhille et al., 2015). Corticosterone (CORT), the final product of the hypothalamic-pituitary-adrenal (HPA) axis in rodents, is recognized as a biomarker of stress (Dallman and Jones, 1973). However, we know that the role of CORT extends into arenas of learning and memory (Sandi et al., 1997), reward-learning (Tomie et al., 2002), and reinforcement (Piazza et al., 1993). Of particular relevance, CORT is involved in forming Pavlovian associations for both aversive (Marchand et al., 2007) and appetitive (Tomie et al., 2002) stimuli (for review, see Lopez and Flagel, 2020). With respect to the latter, relative to GTs, STs show a greater rise in CORT following an initial PavCA session, before the development of a conditioned response (Flagel et al., 2009). Baseline CORT levels before training do not differ between phenotypes (Flagel et al., 2009), but it remains to be determined whether baseline CORT changes as a consequence of cue-reward learning. This was particularly important to assess in the current study, to account for potential learning-induced differences in CORT that may alter stress responsivity on subsequent tests.

In the current study, we assessed baseline CORT levels before and after the acquisition of PavCA behavior (experiment 1A). We hypothesized that any changes in CORT over the course of Pavlovian learning would be a function of the propensity to attribute incentive salience to reward cues. In addition, we assessed CORT and behavioral responses to the elevated plus maze (EPM), open field test (OFT), and acute physiological restraint, to determine whether GTs and STs differ in stress responsivity (experiment 1B). We hypothesized that these phenotypes would not differ in stress reactivity and that any differences in CORT would be specific to Pavlovian learning. Further, to examine a central regulator of stress responsivity (Reul et al., 1987; Akil, 2005), we assessed glucocorticoid receptor (GR) mRNA in the hippocampus and prelimbic cortex (PrL; experiment 2). These brain regions were selected as both are integral to regulation of the stress response (for review, see Herman et al., 2003), and both have been implicated in incentive salience attribution (Fitzpatrick et al., 2016; Campus et al., 2019). Thus, we hypothesized that GR mRNA would differ between phenotypes in the hippocampus and PrL. Together, these studies expand the characterization of the neurobehavioral endophenotype captured by the GT/ST model.

## Materials and Methods

### Experiment 1: general procedures

#### Animals

For experiment 1 (A and B), 60 male Sprague Dawley rats were obtained from Charles River Breeding Labs [Colony 72 (C72) and Colony 04 (R04)]. Rats weighed between 225–275 g on arrival and were pair-housed in standard acrylic cages (46 × 24 × 22 cm) in a temperature-controlled room (22 ± 2°C) under a 12/12 h light/dark cycle (lights on at 7 A.M.). Food and water were available *ad libitum* for the duration of the study. Rats were allowed to acclimate to their colony room and remained undisturbed in their homecages for 7 d after arrival. Rats were then briefly handled every day for five consecutive days before any experimental manipulation. During the last 2 d of handling, 25 45-mg banana-flavored grain pellets (Bio-Serv) were placed inside the homecage, allowing rats to habituate to the food reward used during PavCA training. Behavioral testing occurred during the light cycle (between 10 A.M. and 2 P.M.). All experimental procedures followed *The Guide for the Care and Use of Laboratory Animals* (8th Ed., 2011, National Academy of Sciences).

#### Behavioral testing

##### PavCA training

All PavCA training took place in standard behavioral testing chambers (MED Associates; 20.5 × 24.1-cm floor area, 29.2 cm high) located inside a room with red lighting. The chambers were enclosed in sound-attenuating boxes equipped with a ventilation fan that provided constant air circulation and served as white noise. Each chamber contained a food-cup centered on one of the walls and placed 6 cm above the grid floor. The food-cup was equipped with an infrared beam, and each beam break was recorded as a head entry. Counterbalanced, right or left of the food-cup, was a retractable lever that illuminated upon presentation and was also placed 6 cm above the floor. A force of at least 10 g was necessary to deflect the lever; this deflection was recorded as a “lever contact.” On the opposite wall, a white house light was placed 1 cm from the top of the chamber. House light illumination signaled the beginning of the session and remained on for the duration of the session.

Rats underwent a single pretraining session, where the food-cup was baited with three grain pellets to direct the rats’ attention to the location of the reward. Once placed in the chamber, the house light turned on after 5 min, signaling the beginning of the session. The pretraining session consisted of 25 trials during which the lever remained retracted, and pellets were delivered randomly into the food-cup; one pellet per trial on a variable interval 30 s schedule (range 0–60 s). The total session length was ∼12.5 min.

Following pretraining, or 24 h later, rats underwent a total of five consecutive PavCA training sessions. Each session consisted of 25 trials on a variable interval 90-s schedule (VI 90, range 30–150 s) during which an illuminated lever (conditioned stimulus; CS) was presented for a total of 8 s, and immediately upon its retraction, a grain pellet (unconditioned stimulus; US) was delivered into the adjacent food-cup. Each session lasted ∼40 min.

The following behavioral measures were recorded during each PavCA session: (1) probability to contact (i.e., deflect) the lever upon its presentation (calculated as the number of trials on which a lever contact occurred, divided by the total number of trials); (2) number of lever contacts; (3) latency to contact the lever for the first time; (4) probability to contact (i.e., enter) the food-cup during presentation of the lever (calculated as the number of trials on which a food-cup contact occurred, divided by the total number of trials); (5) number of food-cup contacts during presentation of the lever; (6) latency to first contact the food-cup during presentation of the lever; and (7) number of food-cup entries during the inter-trial interval. These values were then used to calculate three measures of approach behavior that comprise the PavCA index: (1) response bias [(total lever presses – total food-cup entries) ÷ (total lever presses + total food-cup entries)]; (2) probability difference [probability to approach the lever – the probability to enter the food-cup]; (3) latency difference [± (latency to approach the lever – latency to enter the food-cup) ÷ 8]. As previously described (Meyer et al., 2012), PavCA index score was calculated from the average of sessions 4 and 5 using this formula: [(response bias + probability difference + latency difference) ÷ 3]. Scores ranged from +1 to −1; a more positive score indicated a preference for sign-tracking behavior and a negative score for goal-tracking. The cutoffs for phenotype classification were: ≤−0.5 for a GT, ≥0.5 for a ST, and in between −0.5 and 0.5 for an intermediate responder (IR), those that vacillate between the two conditioned responses.

#### CORT

##### Sample collection

**Figure 1.**
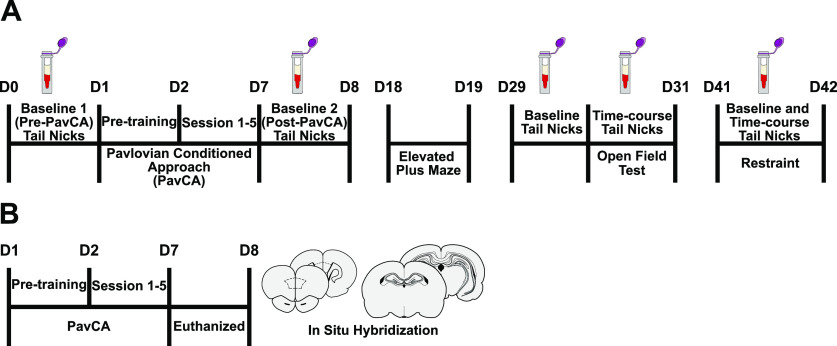
Experimental timelines. ***A***, “Baseline” tail nicks were performed for blood collection Pre-PavCA, and after the rats had acquired a conditioned response (Post-PavCA). Rats were subsequently tested on the elevated plus maze (EPM) and the open field test (OFT), followed by physiological restraint, with a 10-d rest period before each. CORT response to the OFT and acute restraint was captured with time course blood sampling. ***B***, A separate group of rats underwent five sessions of PavCA training and were subsequently euthanized to assess GR expression in the hippocampus and PrL using *in situ* hybridization.

To investigate plasma CORT profiles, blood samples were collected via lateral tail nick at the time points indicated below for experiments 1A and 1B (see also Fig. 1*A*). An experimenter lightly restrained each rat under a blue pad near the edge of a flat surface, allowing their tail to hang off. A small (≤5 mm) nick was made with the tip of a razor blade, and blood was extracted via capillary action (∼200 μl) into an EDTA-coated tube (Sarstedt). Samples were capped, inverted two to three times, and immediately placed onto ice where they remained (<3 h) until the last tail nick was performed. Samples were then separated by centrifugation (13,000 rpm for 10 min at 4°C), and plasma was extracted, flash-frozen on dry ice, and stored at −20°C until processed for radioimmunoassay (RIA).

##### RIA

Plasma CORT levels were measured using commercially available CORT I^125^ double antibody RIA kit (MP Biomedicals) with a minimum detectable dose of 7.7 ng/ml. The manufacturer’s protocol was followed verbatim (and as reported in Clinton et al., 2014). A range of 25–1000 ng/ml CORT calibrators was used to generate a standard logarithmic curve for every set of 76 test tubes (the centrifuge test tube capacity for one spin). For experiments 1A and 1B, a total of 482 plasma samples (not including duplicates or calibration standards) were assayed using 19 centrifuge spins across 6 d, with no more than four sets (i.e., centrifuge spins) per day. Gamma radiation counts per minute were averaged across duplicate samples and converted into CORT concentrations using the average standard curve generated from all sets that were run for each day of RIA. On average, the intra-assay coefficient of variation was 7.24%, while, the interassay coefficient of variation was 16.44%. Outliers were identified and removed if: (1) duplicates had a percent error >10%; or (2) samples were identified as an extreme outlier (3× the interquartile range) by statistical software.

### Experiment 1A: PavCA behavior and baseline plasma CORT profiles

#### CORT

##### Sample collection

Samples were collected, as described above, under baseline conditions before PavCA training (Pre-PavCA) and following the development of a conditioned response to the lever-cue (Post-PavCA; Fig. 1*A*, see experimental timeline). Pre-PavCA tail nicks were performed 24 h before the pretraining sessions (see experiment 1 behavioral testing), while Post-PavCA tail nicks were performed 24 h after the last session (session 5) of training. On days of collection, six rats were transported in their paired-housed homecages into a designated room (start 10:30), where all collection took place under white light. Tail nicks were performed one at a time (∼ 60–90 s per collection). Each wave of six rats remained in the room together but on the opposite side of the room from the collection area. After the last tail nick was performed, all rats were promptly returned to the colony room. Rats were left undisturbed for a total of 10 d before experiment 1B began.

### Experiment 1B: behavioral and CORT response to anxiety-related and stress-related tests in GTs, STs, and IRs

#### CORT

##### Sample collection

Plasma CORT levels induced by behavioral assays of anxiety-like behavior and physiological restraint were captured using tail nick sampling procedures as described above. Collections took place 24 h before the OFT (time 0, or baseline) and 20, 40, 60, and 80 min postonset of the test. For restraint-induced CORT profiles, collections took place immediately when rats were placed into the restraining device (time 0, or baseline) and 30 (before being released), 60, 90, and 120 min after the onset of restraint. Rats were transported into the designated room in a staggered fashion, one at a time to begin collections. Repeated nicks were performed on each rat to capture all of the time points. Up to nine rats remained together in the designated collection room but were on the opposite side of the room from the collection area. Rats were returned to the colony room in a staggered fashion after their last sample was collected.

#### Behavioral testing

##### EPM

After a 10-d rest period following experiment 1A, rats were exposed to an EPM test (Fig. 1*A*, see experimental timeline), considered to be a metric of anxiety-like and risk-taking behavior (Lister, 1987; Walf and Frye, 2007). The apparatus was constructed of four connected arms (each 70 cm from the floor, 45 cm long, and 12 cm wide) made of black Plexiglas and arranged in a cross shape. 45 cm high walls enclosed two opposite arms, and the remaining two were open platforms. A central square (12 × 12 cm) connected all four arms. The test room was dimly lit (40 lux) by a light fixture located above the maze. Before the test, rats were transported inside their homecage, along with their cage mate, into the testing room and left undisturbed to acclimate for 30 min. Upon starting the test, each rat was placed in the central platform facing an open arm and allowed to roam freely around the maze for a total of 5 min. The experimenter remained in the room but was distanced from the apparatus to be out of the rat’s view. A video-tracking system (Noldus Ethovision 11.5) using a live feed from a digital camera mounted on the ceiling directly above the center of the maze was used to detect and record: (1) latency to enter the open arms for the first time; (2) frequency to enter each arm; and (3) time spent in each arm. Additionally, universally used risk assessment behaviors (RABs; see Rodgers et al., 1999; Mikics et al., 2005) were scored manually by the experimenter viewing the live recording. Specifically, the number of times the rat exhibited a bout of grooming, rearing, and protected and unprotected head dips (i.e., head dips over the side of the maze while their body was inside an enclosed arm versus their body being completely exposed on the open platforms) was quantified.

##### OFT

After a 10-d rest period following EPM testing, rats were exposed to an OFT (Fig. 1*A*, see experimental timeline), considered to be another metric of anxiety-like behavior as well as exploratory behavior (Walsh and Cummins, 1976). The OFT test occurred in the same room as the EPM test, and again, paired-housed rats were transported from the colony room to the dimly lit test room and allowed to acclimate for ∼30 min before testing began. The open field apparatus was a 4-wall Plexiglas enclosure with an open top and Plexiglas floor (100 × 100 × 50 cm). At the start of the test, rats were placed into the same corner (bottom left) of the arena and allowed to roam freely for 5 min. Behavior was video recorded with a digital camera mounted above the apparatus. Noldus Ethovision (11.5) was used to detect: (1) the time spent in the center of the arena (a 50 × 50 cm square drawn in the center); (2) the time spent in the outer edge of the arena (25-cm-wide border); (3) the number of entries into the center arena; (4) latency to enter the center of the arena for the first time; and (5) total distance traveled.

##### Restraint

After a 10-d rest period following the OFT, rats underwent a single session of physiological restraint. The restraining device consisted of a white 9 × 12-cm sleeve of flexible Teflon secured with two black Velcro straps attached to a 9 × 3-cm clear Plexiglas platform with a tail slit on one end and breathing holes on the other. Rats were transported in their homecage into the testing room, which was the same as that used for experiment 1A and OFT time course measures. Rats were placed into the restrainer and remained there for 30 min.

### Experiment 2: GR mRNA expression within the hippocampus and PrL of GTs, STs, and IRs

#### Animals

An additional 60 male Sprague Dawley rats were obtained from Charles River Breeding Labs (C72 and R04) for this experiment. Housing and testing conditions were identical to those described in experiment 1, except that lights turned on and off at 6 A.M. and 6 P.M., respectively. Rats were exposed to 2 d of handling before behavioral testing, which occurred between 11 A.M. and 3 P.M.

#### Behavioral testing

##### PavCA training

PavCA training and classification of GTs, STs, and IRs were performed identically to that described above for experiment 1.

#### GR mRNA expression

##### Tissue collection

Twenty-four hours after completion of the fifth PavCA training session (Fig. 1*B*, see experimental timeline), rats underwent live decapitation, and their brains were extracted and immediately flash frozen in 2-methyl butane (−30°C). Brains were stored at −80°C until further processing. Frozen brains were mounted perpendicularly to a metal cryostat chuck using Optimal Cutting Temperature compound (Fisher Healthcare, Thermo Fisher Scientific Kalamazoo) and coated with Shandon M-1 embedding matrix (Thermo Fisher Scientific) in preparation for sectioning. Whole brains were coronally sectioned at 10 μm on a cryostat at −20°C. Brain sections were collected, 4.68 to −7.08 mm from bregma, and directly mounted onto Superfrost Plus microscope slides (Fischer Scientific), with four sections per slide and ∼200 μm between sections on a given slide. Slides were stored at −80°C in preparation for *in situ* hybridization.

##### Probe synthesis

Probes for *in situ* hybridization were synthesized in-house using rat mRNA sequences complementary to the RefSeq database number (M14053) for type II GR (insert size 402, insert location nucleotides 765–1167; identical to García-Fuster et al., 2012). All cDNA segments were extracted using a Qiaquick Gel Extraction kit (QIAGEN), subcloned in Bluescript SK (Stratagene), and confirmed by nucleotide sequencing. The probes were labeled in a reaction mixture of 2 μl of linearized DNA specific to the probe, 10× transcription buffer (Epicentre Technologies Madison), 3 μl of S-35-labeled UTP, 10 μl of S-35-labeled ATP, 1 μl of 10 mm CTP and GTP, 1 μl of 0.1 m dithiothreitol (DTT), 1 μl of RNase inhibitor, and 1 μl of T7 RNA polymerase and incubated for 1.5 h (37°C). Labeled probes were then purified using Micro Bio-Spin 6 Chromatography Column (Bio-Rad), and 1 μl of the probe was counted for subsequent radioactivity dilution calculations. Four to six labelings were used to reach the necessary volume and optimal radioactivity (1–2 million counts per minute/slide). An additional 1 μl of 1 m DTT was also added to the labeled mRNA after purification, allowed to incubate at room temperature for 15 min, and stored at −20°C until further use.

##### *In situ* hybridization

*In situ* hybridization procedures for hippocampal and prelimbic tissue were performed as described below, independently for each brain region. The radioactive probe was diluted in hybridization buffer (50% formamide, 10% dextran sulfate, 3× saline-sodium citrate buffer (SSC), 50 mm sodium phosphate buffer, 1× Denhardt’s solution, 0.1 mg/ml yeast tRNA, and 10 mm DTT) and the volume calculated based on the initial count to obtain roughly 1–2 × 10^6^ radioactivity counts per 75 μl of the diluted probe in hybridization buffer. Slide-mounted brain tissue, 4.68 to 2.52 mm (PrL) and −1.08 to −7.08 mm from bregma (hippocampus), was fixed in 4% paraformaldehyde solution (1 h), washed in 2× SSC, and incubated with 0.1 m triethanolamine (TEA) with 0.25% acetic anhydride (10 min). Slides were then dehydrated using ascending ethanol concentrations and air-dried for 1 h. Hybridization buffer was warmed (37°C) and mixed with the calculated quantity of probe and 1 m DTT (∼1% total HB volume); 75 μl of the diluted probe was then applied to coverslips, which were subsequently placed onto the tissue. Slides were then placed in humidity-maintained hybridization chambers soaked with formamide and incubated overnight (∼16 h) at 55°C. The next day, coverslips were removed, and the slides were rinsed with 2× SSC. Slides were then incubated (1 h) in RNase A solution (100 μg/ml RNase in Tris buffer with 0.5 m NaCl; 37°C), washed in descending concentrations of SSC (2×, 1×, 0.5×), and incubated (1 h) in 0.1× SSC (65°C). Next, sections were briefly rinsed in H_2_O, dehydrated using ascending ethanol concentrations, and air-dried for 1 h. Slides were then loaded into film cassettes, separated by regions of interest (i.e., PrL and hippocampus) and exposed in a dark room to 35 × 43-cm Kodak BioMax MR film (Carestream Health Inc) for three weeks for PrL and seven weeks for hippocampal tissue. Extra slides using spare experimental tissue were run concurrently to confirm optimal exposure time. The specificity of the probe was verified using sense strand controls similar to previous studies (Kabbaj et al., 2000; García-Fuster et al., 2012).

##### Quantification

Films were developed using Microtek ScanMaker 1000XL and digitally scanned using SilverFast Lasersoft Imaging software. Signal expression was quantified using ImageJ (National Institutes of Health), a computer-assisted optical densitometry software. For hippocampus GR mRNA quantification, the brush selection tool (size: 15 pixels) was used to trace the curvilinear subregions of interest (CA1, CA2, CA3, and dentate gyrus) throughout the dorsal (−2.64 to −4.56 mm from bregma) and ventral hippocampus (−4.68 to −6.72 mm from bregma), using the Rat Brain Atlas (Paxinos and Watson, 2007) for guidance (see also Fig. 7*A*). Area (total number of pixels), optical density (darkness of pixels) and integrated optical density (intensity and spread) measurements of the region of interest were taken using a macro that automatically enabled signal above background (3.5× SD) to be determined. The area (unit, 63 pixels/1 mm) and optical density (darkness) were calculated for each of the four hippocampal subregions across a range of 11–21 sections per rat that spanned the dorsal-ventral gradient of the hippocampus. A single value was calculated for each of the hippocampal subregions per rat, by averaging the values of both hemispheres across multiple sections. Further, given that the dorsal and ventral hippocampus are viewed as neuroanatomically and functionally distinct (see Fanselow and Dong, 2010), with the dorsal hippocampus considered to be more involved in cognitive function and the ventral hippocampus in stress and emotion, a single average value was used for dorsal versus ventral subregions and data were graphed and analyzed separately (similar to Romeo et al., 2008).

For PrL GR quantification, the rectangle selection tool (area set to 0.2318; unit, 63 pixels, 1 mm) was used across anterior (A)-posterior (P) levels (4.68–2.52 mm from bregma). Optical density was calculated across a range of 3–10 sections per rat that spanned the different A-P levels of the PrL. A single value was also calculated per rat, by averaging the values of both hemispheres across multiple sections. For both hippocampus and PrL quantification, sections with damaged tissue or artifacts that distorted the region of interest were omitted from analyses. During quantification, the experimenter was blind to the phenotype assignments, and the same experimenter quantified both the hippocampus and PrL.

### Experiments 1 and 2: statistical analysis

Behavioral outcome measures (i.e., PavCA, EPM, OFT), plasma CORT concentrations, and *in situ* hybridization measures (i.e., area and optical density) were analyzed using the Statistical Package for the Social Sciences (SPSS) program version 24.0 (IBM). Linear mixed-effects models were performed for PavCA behavior and neuroendocrine measures (CORT and GR mRNA levels), using the best fit covariance structure with the lowest Akaike’s information criterion for each set of data. Univariate analysis of variance was performed for behavior exhibited during the EPM and OFT and normality was tested using the Shapiro–Wilk test. When dependent variables failed to meet normality, log 10 or square root transformations were conducted, or a Kruskal–Wallis nonparametric test was performed (using StatView, version 5.0, SAS Institute Inc.). Pearson correlations were performed to determine whether there was a significant relationship between baseline CORT levels (Pre-PavCA vs Post-PavCA) and baseline CORT levels and PavCA behavior. Using univariate analysis of variance, the effect of phenotype was assessed with the predicting variable (e.g., change in baseline CORT) set as a covariate. Thus, a significant interaction between the two variables would indicate that the correlational relationship differs between phenotypes. Statistical significance was set at *p* < 0.05, and Bonferroni *post hoc* analyses were conducted when significant interactions were detected. All figures were made using GraphPad Prism 7.

## Results

### Experiment 1: PavCA behavior and baseline plasma CORT profiles

#### PavCA behavior

**Figure 2.**
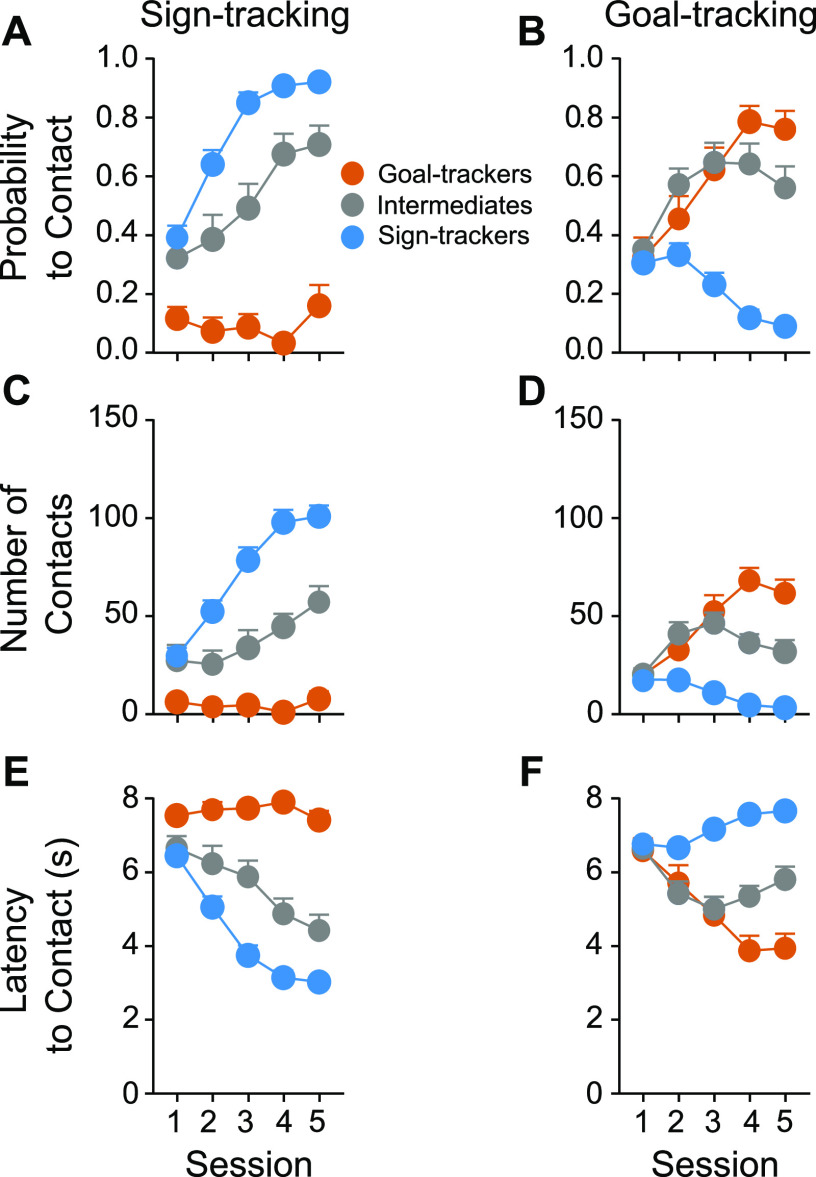
Acquisition of sign-tracking and goal-tracking behavior. Sign-tracking (i.e., lever-directed, left panels) and goal-tracking (i.e., food-cup directed, right panels) behavioral measures were assessed across five PavCA sessions. Mean + SEM for probability to contact (***A***) the lever or (***B***) the food-cup; total number of contacts with (***C***) the lever or (***D***) the food-cup; and latency to contact (***E***) the lever or (***F***) the food-cup. Rats with a sign-tracking conditioned response were classified as STs (*n* = 32), those with a goal-tracking conditioned response as GTs (*n* = 11), and those that vacillated between the two conditioned responses as IRs (*n* = 17).

The following lever-directed (sign-tracking) and food cup-directed (goal-tracking) behaviors were assessed across five consecutive PavCA training sessions and compared between GTs (*n* = 11), IRs (*n* = 17), and STs (*n* = 32): the probability to contact, the number of contacts, and the latency to contact the lever or food-cup during the presentation of the lever-CS (Fig. 2). Main effects of phenotype, session, and phenotype × session interactions for all behavioral measures are reported in Table 1, top. There was a significant effect of phenotype and session for all behavioral measures. As expected, STs showed a significantly greater probability to contact the lever (Fig. 2*A*), a greater number of lever contacts (Fig. 2*C*), and shorter latency to contact the lever (Fig. 2*E*), relative to IRs and GTs. These differences in lever-directed behaviors were apparent by the second PavCA training session (Fig. 2; see also Table 2, top left). In contrast, relative to STs and IRs, GTs showed a significantly greater probability of entering the food-cup (Fig. 2*B*), a greater number of food-cup entries (Fig. 2*D*), and a shorter latency to enter the food-cup (Fig. 2*F*). These differences in food cup-directed behavior became apparent by the third PavCA training session (Fig. 2; also see Table 2, top right).

**Table 1:** Results from linear mixed model analysis for sign-tracking (i.e., lever-directed) and goal-tracking (i.e., food-cup-directed) behaviors

PavCA behavior									
Experiment 1	Number of lever contacts			Probability to contact lever			Latency to contact lever		
Sign-tracking	df_1_,df_2_	*F* value	*p* value	df_1_,df_2_	*F* value	*p* value	df_1_,df_2_	*F* value	*p* value
Effect of phenotype	2,54.615	38.121	*p* < 0.001	2,57	56.803	*p* < 0.001	2,55.352	40.450	*p* < 0.001
Effect of session	4,87.711	15.929	*p* < 0.001	4,57	23.654	*p* < 0.001	4,79.678	35.451	*p* < 0.001
Phenotype × session	8,87.711	8.640	*p* < 0.001	8,57	9.027	*p* < 0.001	8,79.678	11.928	*p* < 0.001
	Number of food cup contacts			Probability to contact food cup			Latency to contact food cup		
Goal-tracking	df_1_,df_2_	*F* value	*p* value	df_1_,df_2_	*F* value	*p* value	df_1_,df_2_	*F* value	*p* value
Effect of phenotype	2,63.287	52.264	*p* < 0.001	2,63.003	39.844	*p* < 0.001	2,60.931	45.807	*p* < 0.001
Effect of session	4,96.698	14.610	*p* < 0.001	4,155.487	10.960	*p* < 0.001	4,81.979	14.380	*p* < 0.001
Phenotype × session	8,96.698	16.953	*p* < 0.001	8,155.487	12.193	*p* < 0.001	8,87.711	17.313	*p* < 0.001
Experiment 2	Number of lever contacts			Probability to lever			Latency to contact lever		
Sign-tracking	df_1_,df_2_	*F* value	*p* value	df_1_,df_2_	*F* value	*p* value	df_1_,df_2_	*F* value	*p* value
Effect of phenotype	2,25.738	73.590	*p* < 0.001	2,27.026	111.836	*p* < 0.001	2,27.037	65.914	*p* < 0.001
Effect of session	4,30.902	17.660	*p* < 0.001	4,25.527	37.054	*p* < 0.001	4,23.863	29.661	*p* < 0.001
Phenotype × session	8,30.902	10.840	*p* < 0.001	8,25.527	17.592	*p* < 0.001	8,23.885	12.406	*p* < 0.001
	
	Probability to Contact food cup			Number of food cup contacts			Latency to contact food cup		
Goal-tracking	df_1_,df_2_	*F* value	*p* value	df_1_,df_2_	*F* value	*p* value	df_1_,df_2_	*F* value	*p* value
Effect of phenotype	2,31.080	39.402	*p* < 0.001	2,27.941	37.563	*p* < 0.001	2,26.445	41.540	*p* < 0.001
Effect of session	4,39.514	5.3831	*p* = 0.002	4,47.772	6.000	*p* = 0.001	4,56.426	9.495	*p* < 0.001
Phenotype × session	8,39.631	7.976	*p* < 0.001	8,47.731	10.586	*p* < 0.001	8,56.453	12.399	*p* < 0.001

Effect of phenotype, session, and phenotype × session interactions were analyzed for experiment 1 (top) and experiment 2 (bottom). df_1_, degrees of freedom numerator; df_2_, degrees of freedom denominator.

**Table 2:** Bonferroni *post hoc* comparisons between phenotypes for each PavCA session

Phenotype comparisons Experiment 1
	Sign-tracking					Goal-tracking				
	Number of lever contacts					Number of food cup contacts				
	1	2	3	4	5	1	2	3	4	5
GT vs IR	*p* = 0.090	*p* = 0.152	*p* = 0.083	*p* = 0.001*	*p* < 0.001*	*p* = 1.000	*p* = 0.820	*p* = 1.000	*p* < 0.001*	*p* < 0.001*
GT vs ST	*p* = 0.024*	*p* < 0.001*	*p* < 0.001*	*p* < 0.001*	*p* < 0.001*	*p* = 1.000	*p* = 0.072	*p* < 0.001*	*p* < 0.001*	*p* < 0.001*
ST vs IR	*p* = 1.000	*p* = 0.007*	*p* < 0.001*	*p* < 0.001*	*p* < 0.001*	*p* = 1.000	*p* < 0.001*	*p* < 0.001*	*p* < 0.001*	*p* < 0.001*
	Probability to contact lever					Probability to contact food cup				
	1	2	3	4	5	1	2	3	4	5
GT vs IR	*p* = 0.074	*p* = 0.018*	*p* < 0.001*	*p* < 0.001*	*p* < 0.001*	*p* = 1.000	*p* = 0.542	*p* = 1.000	*p* = 0.226	*p* = 0.051
GT vs ST	*p* = 0.004*	*p* < 0.001*	*p* < 0.001*	*p* < 0.001*	*p* < 0.001*	*p* = 1.000	*p* = 0.382	*p* < 0.001*	*p* < 0.001*	*p* < 0.001*
ST vs IR	*p* = 0.930	*p* = 0.012*	*p* < 0.001*	*p* < 0.001*	*p* = 0.001*	*p* = 1.000	*p* = 0.002*	*p* < 0.001*	*p* < 0.001*	*p* < 0.001*
	Latency to contact lever					Latency to contact food cup				
	1	2	3	4	5	1	2	3	4	5
GT vs IR	*p* = 0.092	*p* = 0.057	*p* = 0.006*	*p* < 0.001*	*p* < 0.001*	*p* = 1.000	*p* = 1.000	*p* < 0.001*	*p* < 0.001*	*p* < 0.001*
GT vs ST	*p* = 0.011*	*p* < 0.001*	*p* < 0.001*	*p* < 0.001*	*p* < 0.001*	*p* = 1.000	*p* = 0.081	*p* < 0.001*	*p* < 0.001*	*p* < 0.001*
ST vs IR	*p* = 1.000	*p* = 0.047*	*p* < 0.001*	*p* < 0.001*	*p* = 0.001*	*p* = 1.000	*p* = 0.003*	*p* < 0.001*	*p* < 0.001*	*p* < 0.001*

	Sign-tracking					Goal-tracking				
	Number of lever contacts					Number of food cup contacts				
Experiment 2	1	2	3	4	5	1	2	3	4	5
GT vs IR	*p* = 0.852	*p* = 0.005*	*p* = 0.016*	*p* = 0.001*	*p* < 0.001*	*p* = 0.959	*p* = 1.000	*p* = 0.063	*p* < 0.001*	*p* < 0.001*
GT vs ST	*p* = 0.001*	*p* < 0.001*	*p* < 0.001*	*p* < 0.001*	*p* < 0.001*	*p* = 0.073	*p* = 0.052	*p* < 0.001*	*p* < 0.001*	*p* < 0.001*
ST vs IR	*p* = 0.010*	*p* < 0.001*	*p* < 0.001*	*p* < 0.001*	*p* < 0.001*	*p* = 0.547	*p* = 0.217	*p* = 0.001*	*p* < 0.001*	*p* = 0.003*
	Probability to contact lever					Probability to contact food cup				
	1	2	3	4	5	1	2	3	4	5
GT vs IR	*p* = 0.211	*p* < 0.001*	*p* < 0.001*	*p* < 0.001*	*p* < 0.001*	*p* = 1.000	*p* = 1.000	*p* = 0.820	*p* = 0.093	*p* = 0.005*
GT vs ST	*p* < 0.001*	*p* < 0.001*	*p* < 0.001*	*p* < 0.001*	*p* < 0.001*	*p* = 0.163	*p* = 0.138	*p* < 0.001*	*p* < 0.001*	*p* < 0.001*
ST vs IR	*p* = 0.018*	*p* < 0.001*	*p* = 0.004*	*p* = 0.003*	*p* = 0.003*	*p* = 0.331	*p* = 0.359	*p* < 0.001*	*p* < 0.001*	*p* < 0.001*
	Latency to contact lever					Latency to contact food cup				
	1	2	3	4	5	1	2	3	4	5
GT vs IR	*p* = 0.649	*p* = 0.005*	*p* < 0.001*	*p* < 0.001*	*p* < 0.001*	*p* = 1.000	*p* = 1.000	*p* = 0.072	*p* < 0.001*	*p* < 0.001*
GT vs ST	*p* < 0.001*	*p* < 0.001*	*p* < 0.001*	*p* < 0.001*	*p* < 0.001*	*p* = 0.285	*p* = 0.096	*p* < 0.001*	*p* < 0.001*	*p* < 0.001*
ST vs IR	*p* = 0.013*	*p* = 0.001*	*p* < 0.001*	*p* < 0.001*	*p* = 0.002*	*p* = 0.420	*p* = 0.408	*p* = 0.006*	*p* < 0.001*	*p* < 0.001*

Sign-tracking (i.e., lever-directed) and goal-tracking (i.e., food-cup-directed) behaviors are included for experiment 1 (top) and experiment 2 (bottom). *p < 0.005.

#### Baseline CORT levels Pre-PavCA and Post-PavCA

##### CORT levels

**Figure 3.**
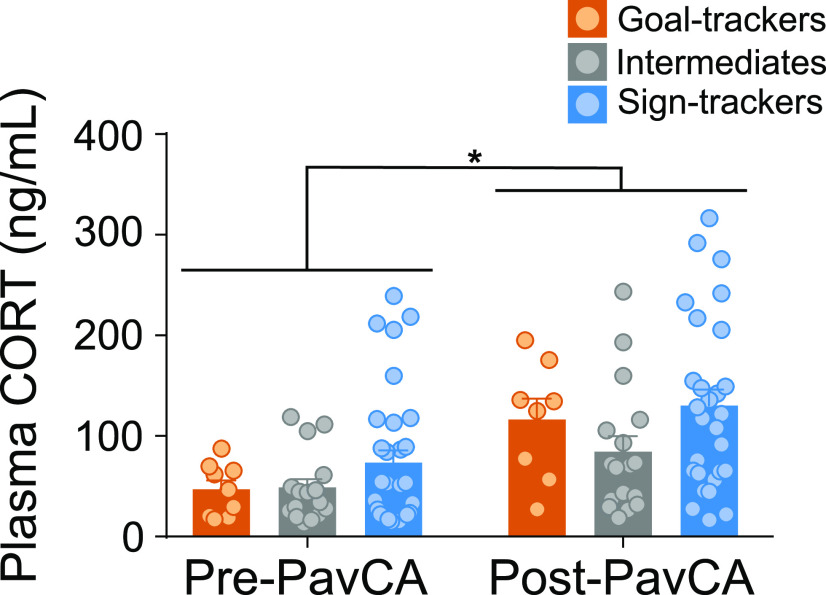
“Baseline” CORT levels before and after PavCA training. Mean + SEM for baseline plasma CORT levels before (Pre-PavCA) and following (Post-PavCA) PavCA training experience. Basal plasma CORT levels increased with Pavlovian training experience (*p = 0.001; *n* = 60; GT *n* = 10, IR = 17, ST = 32).

Overall, Pre-PavCA and Post-PavCA baseline plasma CORT levels did not significantly differ between phenotypes (GTs *n* = 10, IRs *n* = 17, STs *n* = 32; effect of phenotype: *F*_(2,57.691)_ = 2.325, *p *=* *0.107; Fig. 3). Relative to Pre-PavCA, Post-PavCA baseline CORT levels were significantly higher (effect of timepoint: *F*_(1,53.246)_ = 20.180, *p *<* *0.001), rising from an overall average of 56 ng/ml (Pre-PavCA) to 108 ng/ml (Post-PavCA). While baseline CORT levels appear to rise with the experience of PavCA training, the extent to which CORT increased was not dependent on phenotype (time point × phenotype interaction: *F*_(2,52.633)_ = 0.535, *p *=* *0.589). These data are in agreement with prior studies (Tomie et al., 2000; Flagel et al., 2009), demonstrating that Pre-PavCA baseline plasma CORT levels do not significantly differ between phenotypes, and extend these findings to show that baseline plasma CORT levels also do not differ between phenotypes after the development of a conditioned response.

##### Correlations

To further investigate the relationship between baseline CORT levels and cue-motivated behavior, we performed correlational analyses. Pre-PavCA baseline levels significantly correlated with Post-PavCA baseline levels (*r* = 0.449, *p *=* *0.001), but Pre-PavCA baseline levels did not correlate with the behavioral phenotype that emerges with PavCA training (i.e., the average PavCA index from sessions 4 and 5; *r* = 0.198, *p *=* *0.143). In relation, there was not a significant correlation between the change in baseline CORT levels from Pre-PavCA to Post-PavCA (i.e., Δ CORT) and the magnitude of change in the conditioned response from the onset of training (session 1) to the end of training (session 5; i.e., Δ PavCA index; *r* = 0.121, *p *=* *0.402; Table 3). While these data provide little evidence of a relationship between baseline CORT levels and the propensity to attribute incentive salience to a reward cue, prior findings (Flagel et al., 2009) prompted us to further assess this relationship within each phenotype group. When analyzed independently, there is a significant positive correlation between Δ CORT and Δ PavCA index for STs (*r* = 0.470, *p *=* *0.013), and a non-significant negative correlation in GTs (*r* = −0.301, *p* = 0.512) and IRs (*r* = −0.058, *p = 0.*830). Notably, these results should be interpreted with caution as there was not an interaction with the predicting variable (phenotype × Δ CORT: *F*_(2,4)_ = 1.858, *p *=* *0.168) to suggest that this relationship significantly differs between phenotypes, and the sample size for GTs is quite low. Nonetheless, it should also be noted that a similar pattern holds true for the relationship between Post-PavCA CORT and Δ PavCA index, with significant correlations between CORT values and behavioral measures for STs, but not for GTs or IRs (see Table 3).

**Table 3:** Results of Pearson correlation analysis between the change in the PavCA index from session 1 to session 5 (Δ PavCA index) and baseline CORT profiles (Pre-PavCA, Post-PavCA, and the change in baseline values, Δ CORT) for the entire population (i.e., all) and for each phenotype separately

CORT and behavior correlations										
		Pre-PavCA CORT levels			Post-PavCA CORT levels			Pre- to Post-PavCA CORT level Difference “Δ CORT”		
		*n* =	*r*-value	*p*-value	*n* =	*r*-value	*p*-value	*n* =	*r*-value	*p*-value
“Δ PavCA Index”
	All	56	0.262	0.051	53	0.290	0.035*	50	0.121	0.402
	GTs	9	0.667	0.045*	8	0.023	0.957	7	–0.301	0.512
	IRs	16	0.422	0.103	17	0.151	0.563	16	−0.058	0.830
	STs	31	0.058	0.768	28	0.437	0.020*	27	0.470	0.013*

*p < 0.005

### Experiment 1B: behavioral and CORT response to anxiety-related and stress-related tests in GTs, STs, and IRs

#### EPM

**Figure 4.**
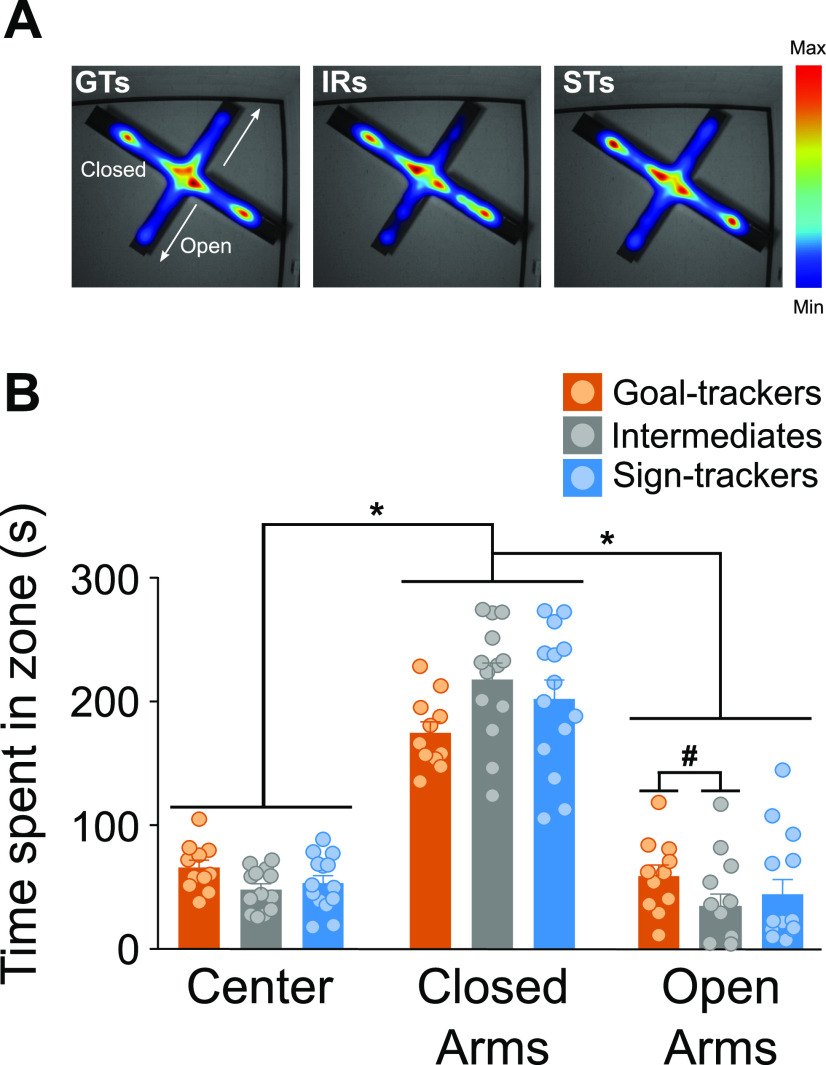
EPM. ***A***, Heat map representations for the average time spent in each zone during the 5-min EPM test for each phenotype. ***B***, Mean + SEM for the time spent in each zone of the EPM for GTs (*n* = 11), IRs (*n* = 13), and STs (*n* = 14). All rats spent significantly more time in the closed arms compared with the open arms and center of the maze (*p < 0.001). There was not a significant difference between GTs and STs in the amount of time spent in either the center of the arena or the open or closed arms. IRs spent significantly less time in the open arms, relative to GTs (#*p* < 0.05).

GTs (*n* = 11) and STs (*n* = 14) did not significantly differ on any behavioral outcome measure of the EPM test, but statistical analysis revealed significant differences relative to their IR counterparts (*n* = 13). While all rats spent the most time (effect of zone: *F*_(2,105)_ = 140.397, *p *<* *0.001) inside the closed arms ($\bar{x}$= 51.21%), relative to the open arms ($\bar{x}$= 21.91%) or center square ($\bar{x}$ = 26.88%), IRs spent significantly less time (phenotype × zone interaction: *F*_(4,105)_ = 2.762, *p *=* *0.031) inside the open arms, relative to GTs (*p *=* *0.011; Fig. 4*B*). However, the latency to enter the open arms for the first time was similar across all phenotypes (effect of phenotype: *F*_(2,35)_ = 0.187, *p *=* *0.83; data not shown) and, in general, relative to IRs, the extreme phenotypes (GTs, *p *=* *0.02, and STs, *p *=* *0.049) entered different zones of the EPM more frequently (effect of phenotype: *F*_(2,105)_ = 6.744, *p *=* *0.002; data not shown). Additionally, there were no significant differences between phenotypes for any of the RABs during the EPM test: frequency of grooming (Kruskal–Wallis test, effect of phenotype: χ^2^_(2)_ = 1.984, *p *=* *0.371), rearing (effect of phenotype: *F*_(2,35)_ = 2.232, *p* = 0.122), protected head dips (effect of phenotype: *F*_(2,35)_ = 0.496, *p *=* *0.613), or unprotected head dips (effect of phenotype: *F*_(2,24)_ = 0.207, *p *=* *0.814; data not shown).

#### OFT

**Figure 5.**
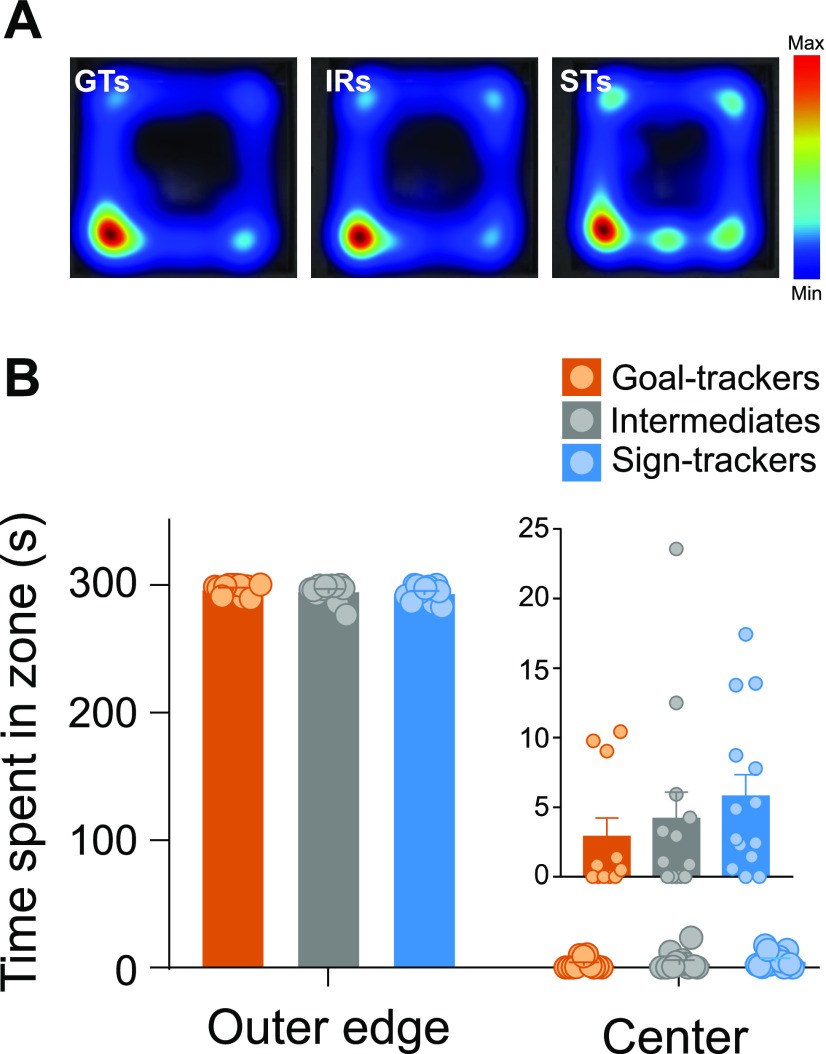
OFT. ***A***, Heat map representations for the average time spent in each zone (outer edge vs center) during the 5-min OFT for each phenotype. ***B***, Mean + SEM for time spent in the outer edge or center of the arena for GTs (*n* = 11), IRs (*n* = 13), and STs (*n* = 14). All rats spent significantly more time on the outer edge of the arena compared with the center. Time spent in the center of the arena is shown as an inset on a different scale for illustration purposes. There was not a significant difference between phenotypes for the amount of time spent in the center of the arena.

There were no significant differences between phenotypes in their behavior on the OFT. All rats spent a comparable amount of time in the outer edge of the arena (Kruskal–Wallis, effect of phenotype: χ^2^_(2)_ = 2.012, *p *=* *0.366), with little time spent in the center of the arena (Fig. 5). There were no significant differences in the number of entries to the center of the arena (Kruskal–Wallis effect of phenotype: χ^2^_(2)_ = 3.029, *p *=* *0.220; data not shown), latency to enter the center of the arena (Kruskal–Wallis effect of phenotype: χ^2^_(2)_ = 2.345, *p *=* *0.310; data not shown), or time spent in the center of the arena (Kruskal–Wallis, effect of phenotype: χ^2^_(2)_ = 2.053, *p *=* *0.358; Fig. 5). The distance traveled during the OFT was also similar between phenotypes (Kruskal–Wallis effect of phenotype: χ^2^_(2)_ = 3.287, *p *=* *0.193; data not shown). These data suggest that STs, GTs and IRs do not differ in anxiety-like behavior, which is consistent with the data described above from the EPM.

#### CORT response

##### CORT response to OFT

**Figure 6.**
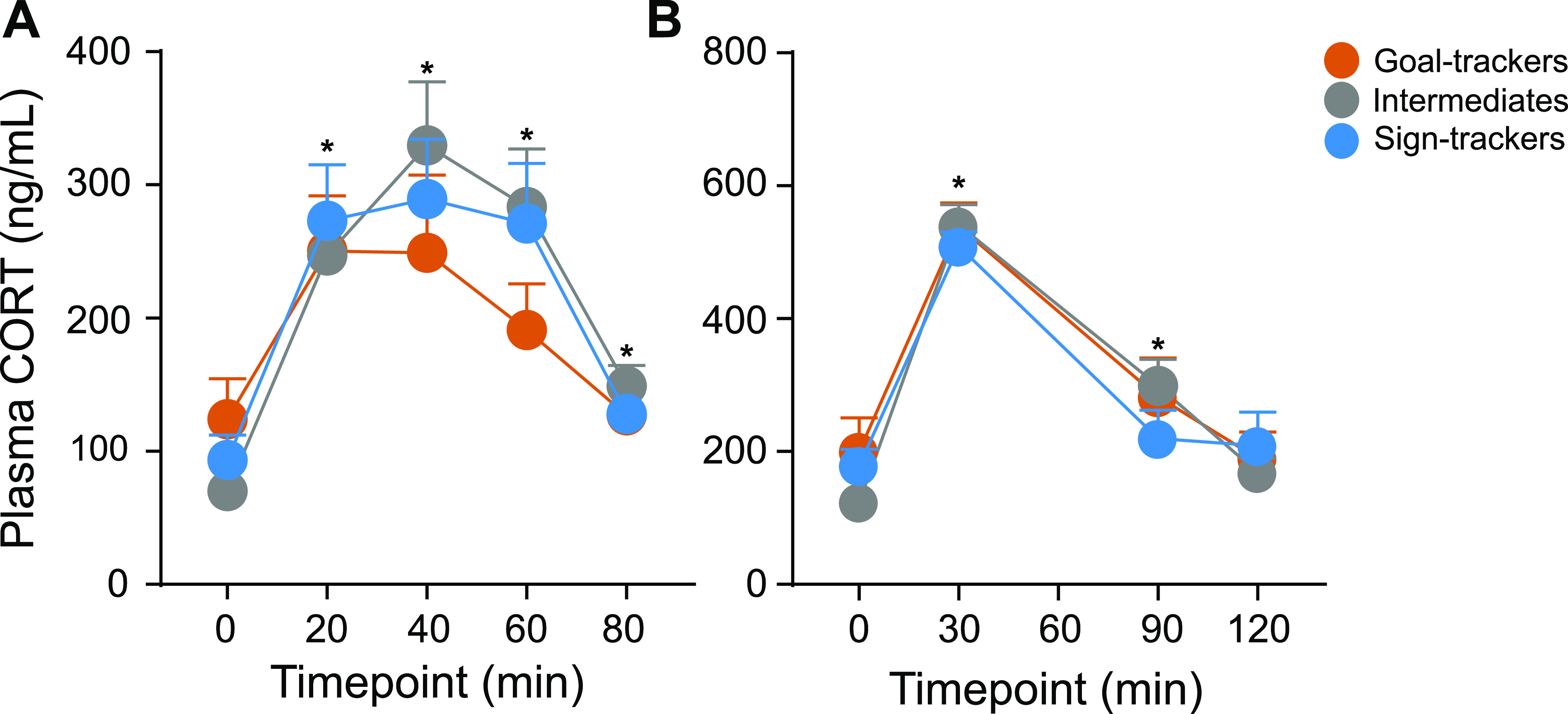
CORT response to the OFT and acute physiological restraint. ***A***, Mean + SEM for plasma CORT levels 0, 20, 40, 60, and 80 min postonset of the OFT for GTs (*n* = 11), IRs (*n* = 13), and STs (*n* = 14). There was a significant increase in CORT induced by the OFT at 20-, 40-, 60-, and 80-min time points (*p < 0.001), but no significant difference between phenotypes. ***B***, Mean + SEM for plasma CORT levels 0, 30, 90, and 120 min postonset of acute restraint for GTs (*n* = 11), IRs (*n* = 13), and STs (*n* = 12). There was a significant increase in CORT induced by restraint at 30- and 90-min time points (*p < 0.001), but no significant difference between phenotypes.

Exposure to the OFT elicited a CORT response (effect of time: *F*_(4,44.652)_ = 12.849, *p *<* *0.001), with a significant rise relative to baseline at 20, 40, 60, and 80 min post-OFT onset. Although the CORT response was decreased at 80 min relative to the peak response (40 vs 80 min, *p *<* *0.001), a return to baseline levels was not captured with this time course (baseline vs 80 min, *p* = 0.041). Nonetheless, the CORT response to the OFT did not significantly differ between phenotypes (effect of phenotype: *F*_(2,35.564)_ = 0.215, *p *=* *0.808; time × phenotype interaction: *F*_(8,45.180)_ = 0.718, *p *= 0.675; Fig. 6*A*).

##### CORT response to physiological restraint

Acute physiological restraint (30 min) elicited a CORT response (effect of time: *F*_(3,28.058)_ = 157.308, *p *<* *0.001), with a significant rise relative to baseline at 30 and 90 min, and return to baseline levels at 120 min postonset of restraint. There was not a significant difference in the CORT response to acute restraint between phenotypes (effect of phenotype: *F*_(2,32.084)_ = 0.114, *p *=* *0.893; time × phenotype interaction: *F*_(6,29.646)_ = 1.568, *p *=* *0.191; Fig. 6*B*).

### Experiment 2: GR mRNA expression within the hippocampus and PrL of GTs, STs, and IRs

#### PavCA behavior

Similar to experiment 1, there were significant effects of phenotype, session, and phenotype × session interactions for all behavioral measures reported in Table 1, bottom (data are not shown in graphical format). Differences between phenotypes were apparent for lever-directed and food cup-directed behavior as early as the first PavCA training session (see Table 2, bottom).

#### GR mRNA expression

##### Dorsal hippocampus

**Figure 7.**
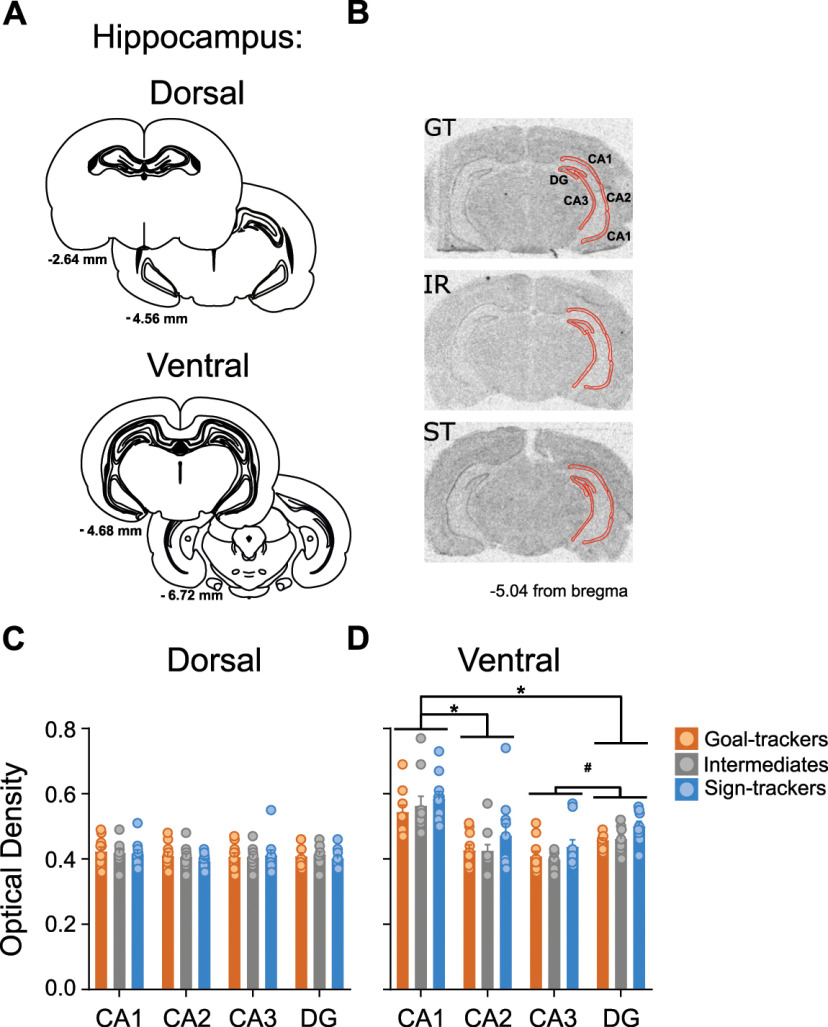
GR mRNA expression in the dorsal and ventral hippocampus. ***A***, Coronal brain sections representing bregma coordinates used to quantify GR mRNA expression (adapted from Paxinos and Watson, 2007). ***B***, Representative *in situ* images for a GT, IR, and ST rat with tracing selections of the region of interest (ROI; in red) on the right hemisphere, including hippocampal subregions demarcated as CA1, CA2, CA3, and DG. ***C***, ***D***, Mean + SEM optical density for GR mRNA in subregions of the (***C***) dorsal and (***D***) ventral hippocampus for GTs (*n* = 10), IRs (*n* = 10) and STs (*n* = 10). In the ventral hippocampus, GR mRNA varied between subregions (*p < 0.001 vs CA1, #*p* < 0.001 vs DG). Relative to GTs and IRs, STs show greater GR mRNA density across subregions.

There were no significant differences between phenotypes in GR mRNA expression (i.e., optical density) in the dorsal hippocampus (effect of phenotype: *F*_(2,108)_ = 0.233, *p *=* *0.793), and no significant difference in expression patterns between subregions of the dorsal hippocampus (effect of subregion: *F*_(3,108)_ = 1.089, *p *=* *0.357; Fig. 7*C*). Given the anatomic variability in size between subregions (Fig. 7*A*, schematic), significant differences in area were detected (effect of subregion: *F*_(3,108)_ = 1020.291, *p *<* *0.001; data not shown); CA1 subregion contained the largest area ($\bar{x}$ = 1.132), while CA2 the smallest ($\bar{x}$ = 0.147). However, the regions of interest were manually outlined (CA1, CA2, CA3, dentate gyrus (DG)), and the area was not dependent on phenotype (effect of phenotype: *F*_(2,108)_ = 0.118, *p *=* *0.889; phenotype × subregion interaction: *F*_(6,108)_ = 0.417, *p *=* *0.866), indicating that the selection of regions of interest was consistent across phenotypes.

##### Ventral hippocampus

Unlike the dorsal hippocampus, GR mRNA expression significantly differed between phenotypes (effect of phenotype: *F*_(2,108)_ = 4.601, *p *=* *0.012) and subregions (effect of subregion: *F*_(3,108)_ = 30.464, *p* < 0.001) in the ventral hippocampus. STs ($\bar{x}$= 0.497) had greater optical density relative to GTs ($\bar{x}$ = 0.458, *p *= 0.022) and IRs ($\bar{x}$= 0.461, *p *=* *0.040), and there was greater optical density in CA1 ($\bar{x}$= 0.563) relative to CA2 ($\bar{x}$ = 0.441, *p *<* *0.001), CA3 ($\bar{x}$= 0.413, *p *<* *0.001), and DG ($\bar{x}$ = 0.472, *p *<* *0.001; Fig. 7*D*). In addition, optical density in DG was greater than that in CA3 (*p *=* *0.004). Like the dorsal hippocampus, area was significantly different between subregions (subregion: *F*_(3,108)_ = 172.935, *p *<* *0.001), but not between phenotypes (effect of phenotype: *F*_(2,108)_ = 0.579, *p *=* *0.562; phenotype × subregion interaction: *F*_(6,108)_ = 0.876, *p *=* *0.515; data not shown).

##### PrL

**Figure 8.**
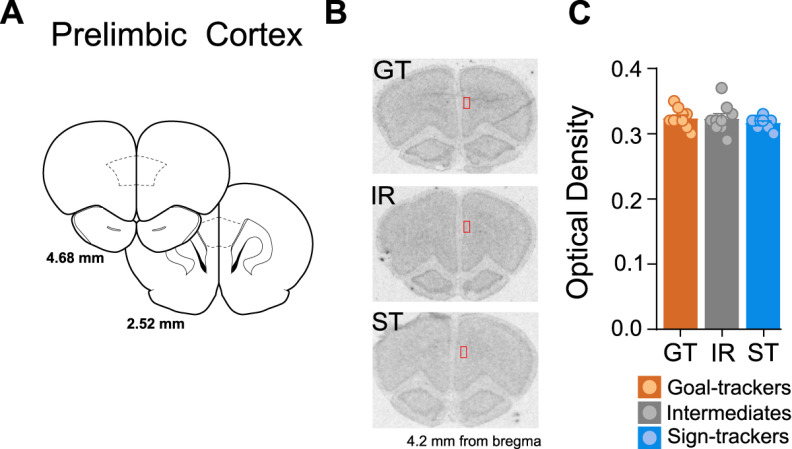
GR mRNA expression in the PrL. ***A***, Coronal brain sections representing bregma coordinates used to quantify GR mRNA expression (adapted from Paxinos and Watson, 2007). ***B***, Representative *in situ* images for a GT, IR, and ST rat with tracing selections of the region of interest (ROI; in red) on the right hemisphere. ***C***, Mean + SEM optical density for GR mRNA in the PrL for GTs (*n* = 10), IRs (*n* = 10), and STs (*n* = 10).

Given GR mRNA expression within the PrL did not differ between A-P levels (effect of A-P level: *F*_(6,146)_ = 0.191, *p* = 0.979; A-P level × phenotype interaction: *F*_(12,146)_ = 0.298, *p *=* *0.989), GR mRNA values were averaged per rat across all A-P levels. GR mRNA expression did not differ between phenotypes in the PrL (effect of phenotype: *F*_(2,26)_ = 0.811, *p *=* *0.455; Fig. 8*C*).

## Discussion

The present studies examined whether differences in behavioral and neuroendocrine measures of stress and anxiety are included among the co-existing traits associated with the propensity to attribute incentive salience to reward cues. We report three main findings. First, there is a general increase in baseline plasma CORT levels over the course of associative cue-reward learning, and this increase is independent of the Pavlovian conditioned response that emerges. Second, behavioral and CORT responses to environmental challenges that elicit aversive arousal do not differ between GTs and STs. Third, STs have greater expression of GR mRNA in the ventral hippocampus relative to GTs and IRs, whereas GR mRNA in the dorsal hippocampus and PrL is comparable between phenotypes.

The basis of our understanding of CORT function in appetitive Pavlovian conditioning stems from the work of Tomie et al. (2000), who demonstrated that cue-food associations elicit an increase in plasma CORT. Subsequently, it was shown that, relative to GTs, STs exhibit a greater rise in plasma CORT following a single Pavlovian conditioning session; that is, before the development of a conditioned response (Flagel et al., 2009). Further, before PavCA training, baseline CORT is similar across GTs, IRs, and STs (Flagel et al., 2009). To date, however, baseline plasma CORT had not been systematically assessed in the same rat to determine whether CORT profiles change as a consequence of cue-reward learning. Thus, in experiment 1A we compared, within the same rat, baseline CORT concentrations at a “naive” state of learning (Pre-PavCA) and once a conditioned response had been acquired (Post-PavCA). There was a significant rise in baseline plasma CORT levels with the development of a conditioned response, and Pre-PavCA CORT levels correlated with Post-PavCA CORT levels. Contrary to our hypothesis, however, this rise in baseline CORT was not dependent on the innate cue-learning strategy that was employed, as levels did not significantly differ between GTs and STs. Further, baseline CORT levels at a naive state of Pavlovian learning did not predict the behavior that emerged with Pavlovian training, and the change in baseline CORT did not significantly correlate with the change in PavCA behavior over the course of learning. When phenotypes were assessed independently, however, the change in baseline CORT levels was significantly correlated with the change in PavCA behavior in STs, and this relationship was not significant in GTs or IRs. While these correlational data do not prompt strong conclusions in and of themselves, they align with prior reports demonstrating a relationship between CORT and the development of sign-tracking behavior (Tomie et al., 2000; Flagel et al., 2009).

One of the primary roles of CORT is to act across the body and brain to broadly mediate the stress response (Herman et al., 2016). Thus, we wanted to determine whether differences in plasma CORT are present in GTs versus STs in contexts outside of Pavlovian conditioning and, explicitly, in response to paradigms related to stress and anxiety. As we hypothesized, experiment 1B showed no differences between phenotypes in CORT response to an OFT or physiological restraint. Further, GTs and STs did not differ in their behavioral response to the EPM or OFT. Notably, rats spent little time within the center of the open field arena, which may be indicative of especially aversive conditions that could have precluded group differences. Nonetheless, these findings are consistent with those previously reported by Vanhille et al. (2015), who showed no preexisting differences in behavior on the EPM test in rats that were later characterized as STs or GTs; and those reported by Harb and Almeida (2014) who showed no differences in behavior on the OFT in mice characterized as STs or GTs. In contrast to the present findings, however, Harb and Almeida (2014) did report a significant difference in peak CORT response following an acute stressor, with ST mice exhibiting a greater peak relative to GTs or IRs. These discrepant findings are likely because of differences in experimental procedures, including the species used and the nature and intensity of the stressor. In this regard, we note that the repeated testing implemented in the current study may have affected the CORT response in a manner that precluded observable differences (Dallman et al., 2004). Indeed, it is possible that differences in the CORT profile in response to physiological restraint were not apparent because of a ceiling effect, as both baseline and peak CORT levels were high across all animals. Taken together, given that GTs and STs behaved similarly on both tests of anxiety-like behavior, and showed no significant differences in CORT response to either the OFT or physiological restraint, we conclude, based on the paradigms implemented here, that individual differences in aversive arousal are not captured by the GT/ST animal model.

It is important to note that other indices of aversive arousal, including fear conditioning and the associated freezing response have been reported to differ between GTs and STs (Morrow et al., 2011, 2015). Specifically, relative to GTs, STs are more fearful of discrete cues that predict footshock (Morrow et al., 2011), and show exaggerated incubation of their fear response (Morrow et al., 2015). However, GTs exhibit greater contextual fear when placed back into a fear-conditioning context in the absence of discrete cues (Morrow et al., 2011). Thus, these differences seem to be specific to learning the value of the discrete cue, rather than differences in aversive arousal or stress reactivity. Others have shown that CORT plays a critical role in fear conditioning, beyond the stress component (Zorawski and Killcross, 2002; Marchand et al., 2007), but this has yet to be assessed within the context of the GT/ST animal model and will be the focus of future investigations.

While the current findings and those of others (Harb and Almeida, 2014; Vanhille et al., 2015) demonstrate that GTs and STs respond similarly to behavioral assays indicative of stress and anxiety, exposure to stress has been shown to alter the propensity to attribute incentive salience to reward cues (Lomanowska et al., 2011; Hynes et al., 2018; Fitzpatrick et al., 2019). Rats exposed to stress early in life exhibit greater sign-tracking behavior in adulthood (Lomanowska et al., 2011; Hynes et al., 2018). In contrast, adult rats exposed to a single prolonged stressor show an attenuation of sign-tracking behavior (Fitzpatrick et al., 2019). Thus, the impact of stress on the propensity to sign-track appears to be dependent on the type of stressor and timing of exposure. In light of the current findings, we postulate that the neural processes underlying these reported stress-induced effects (Lomanowska et al., 2011; Hynes et al., 2018; Fitzpatrick et al., 2019) go beyond CORT and the HPA axis, and include components of the cortico-thalamic-striatal “motive” circuit, which is differentially engaged in STs versus GTs (Flagel et al., 2011a; see also Kuhn et al., 2018).

The hippocampus (Maccari et al., 1991; for review, see Barr et al., 2017) and prefrontal cortex (Deroche-Gamonet et al., 2003; Butts and Phillips, 2013) are two brain regions that may serve as a potential neural interface between the stress and the motive circuitry. GRs are densely expressed within the hippocampus (Reul and de Kloet, 1985) and CORT-GR interactions within this brain region play a critical role in the negative feedback system that acts to maintain homeostatic levels of CORT in the face of physiological or environmental challenges (Herman et al., 2012). Specifically, greater GR mRNA expression in the hippocampus has been associated with more rapid negative feedback, or return to baseline CORT levels (Meaney et al., 1996; Liberzon et al., 1999). In the current study, we did not observe phenotypic differences in circulating CORT levels either under baseline conditions or in response to environmental challenges (i.e., OFT or physiological restraint). However, we did find that, relative to GTs and IRs, STs have significantly greater expression of GR mRNA in the ventral hippocampus. Although we did not hypothesize phenotypic differences in GR expression to be specific to the ventral hippocampus, the fact that these differences are not apparent in the dorsal hippocampus may explain why we did not observe differences in circulating levels of CORT. Indeed, while the dorsal hippocampus has been shown to play a role in stress-induced negative feedback (Feldman and Weidenfeld, 1993, 1999), the ventral hippocampus has been reported to regulate tonic levels of CORT (Herman et al., 1992). Other findings suggest that the engagement of the ventral versus dorsal hippocampus is dependent on the type of stressor (Herman et al., 2005; Maggio and Segal, 2007; Dorey et al., 2012). Thus, additional studies are warranted to further investigate the role of GR expression in the ventral hippocampus within the context of the stress response and negative feedback regulation. Nonetheless, we propose that the phenotypic differences reported here in GR expression in the ventral hippocampus are directly related to motivated behavior and reward learning, rather than stress regulation. Further, lesions of the ventral, but not the dorsal, hippocampus decrease the propensity to sign-track (Fitzpatrick et al., 2016). While it is remains to be determined whether these lesion effects are dependent on GR function, it should be noted that systemic administration of a GR antagonist similarly attenuates the acquisition of sign-tracking behavior (Rice et al., 2018, 2019).

Like the hippocampus, the prefrontal cortex has inhibitory effects on HPA axis activity (for review, see Herman et al., 2003). Specifically, GRs within the prelimbic (PrL) subregion of the prefrontal cortex are implicated in response to acute stressors, with their absence increasing the CORT response (McKlveen et al., 2013). GRs within the PrL also influence reward-related mediators, like dopamine (Butts and Phillips, 2013). Within the context of the GT/ST model, the PrL is recognized as an integral component of top-down control over incentive salience attribution (Campus et al., 2019), with STs deficient in cortical control, relative to GTs (for review, see Sarter and Phillips, 2018). Given that GTs and STs differ in cortical control (Paolone et al., 2013), plasma CORT response (Flagel et al., 2009), and dopamine response to Pavlovian cues, including within the PrL itself (Flagel et al., 2011b; Pitchers et al., 2017), we expected to observe phenotypic differences in GR mRNA expression in the PrL. Yet, no significant differences were apparent. It should be noted, however, that GR mRNA expression may not be reflective of receptor (i.e., protein) levels. Further investigation is warranted to determine whether GR function in either the hippocampus or PrL plays a role in incentive motivational processes. It is also possible that differences would have been apparent had GR mRNA been assessed in specific types of neurons (e.g., tyrosine hydroxylase-positive or dopamine receptive) or within a given circuit (e.g., PrL-nucleus accumbens). Ongoing studies are investigating this possibility. With these caveats in mind, the current data suggest that GRs, particularly within the ventral hippocampus, play a role in incentive motivational processes.

In conclusion, these findings establish that the neurobehavioral endophenotype associated with the propensity to sign-track does not include differences in stress reactivity. Further, we provide additional evidence that glucocorticoids, which have primarily been implicated in aversive arousal (but see Deroche et al., 1993; Piazza et al., 1993), are also involved in appetitive motivation and, specifically, Pavlovian learning. In addition, expression of GRs in the ventral hippocampus appears to be related to inherent cue-reward learning strategies. As these studies were limited to male rats, the role of CORT in cue-reward learning in females, and stress reactivity in female GTs and STs, warrants further investigation. Together, the findings reported here provide a foundation for future work to further examine the mechanism by which glucocorticoids interact with other neural systems to influence incentive motivational processes (for discussion, see Lopez and Flagel, 2020).

## Synthesis

Reviewing Editor: Laura Bradfield, University of Technology Sydney Decisions are customarily a result of the Reviewing Editor and the peer reviewers coming together and discussing their recommendations until a consensus is reached. When revisions are invited, a fact-based synthesis statement explaining their decision and outlining what is needed to prepare a revision will be listed below. The following reviewer(s) agreed to reveal their identity: Matthew Wanat.

The reviewers and I have now read and discussed your manuscript. Overall, our reactions to your manuscript were mixed, although we all noted that the methodologies employed were sound and the paradigms used were elegant. Nevertheless, there are some concerns about whether the manuscript offers enough of a conceptual advance for publication in eNeuro. I have some suggestions about how these concerns might be addressed.

First, you have several instances here where you report negative results as well as ‘new results’. It is important to note that eNeuro is committed to publishing negative results. From the eNeuro advice to authors it state that research article submission types include:

Negative Results: Research papers from authors who tried to test important hypotheses but did not get the outcome they expected. These manuscripts must include testing the hypothesis by multiple experimental approaches, rigorously reproducing the refuted experimental models, and meticulous use of both positive and negative controls.

I think that your manuscript fulfils most of these criteria, however I think it is important throughout to explicitly highlight the specific hypotheses you were testing that were not borne out by the data (including clear and solid rationales for those hypotheses), as well as discussion as to why CORT/behaviour (for example) did not differ between phenotypes. Indeed you already include some discussion of this, so just some reframing in terms of the hypotheses is necessary, with explicit statements that the hypotheses were not confirmed. Please also keep in mind that the failure to reject the null hypothesis does not necessarily mean that a population difference does not exist, just that it was not detected here.

Second, reviewer #2 and I feel that it would add much interest to the paper if you could include analysis of glucocorticoid receptor mRNA expression in regions outside hippocampus, in particular, medial prefrontal cortex or amygdala. Again, even negative results could be of interest. If there are major practical issues with providing such data however, particularly given the ongoing COVID19 pandemic, please do not hesitate to let me know.

Third, both reviewers and I had some issues with your interpretations of the correlations in Figures 3B and 3C. Please include the relevant statistical analysis (showing that ST correlations significantly differ from that of other phenotypes) or omit the claims about ST differences from other phenotypes according to this measure.

Fourth, if there is any rationale as to why only male rats were used, please include this in the ‘Animals’ section on page 94. Please also include a statement in the discussion that the results may not apply to females, or something to that effect.

Finally, I found some of the asterisks in some of the figures difficult to interpret. For instance, in Figure 4 there is a line above the three phenotypes in the ‘closed arm’ panel and on first glance I thought this indicated a difference between phenotypes. Upon reading the text I realised that this difference is averaged over phenotypes and compared to performance on the open arms. I had similar difficulties throughout, compounded by the fact that the specific parts of the figure was not always referred to in text (e.g. ‘Figure 4’ was referred rather than ‘Figure 4B’). Moreover, the colour of the correlation line for all phenotypes collapsed in Figure 3C is the same as the colour for goal-trackers alone in other Figures. Please make sure that colours are unique and consistent for each phenotype, and that it is clear from the figures exactly what comparison each asterisk is referring to.

The reviews are attached.

Reviewer #1

This manuscript examined if the heterogeneity of Pavlovian conditioned approach behaviors mapped onto corresponding differences in corticosterone levels, glucocorticoid receptor mRNA, and anxiety-like behaviors. The authors demonstrate that anxiety-like behavior was no different between ST, GT, and IR categorized rats. In contrast, there were a notable difference in glucocorticoid receptor expression levels in the ventral hippocampus. The experiments were well-executed and the findings are of interest to a general audience. I have a few concerns that can be easily addressed without the need for further experimentation.

1) The correlations in Fig. 3B-C using all the subjects are appropriate. But I cannot find a justification why the within-group correlational analysis is only performed for the STs. The comparison of r values between these 2 correlational analyses (all rats vs ST only) is descriptive and not supported by statistical analyses. There are 2 potential solutions: (1) include the within-group correlations for IR and GT or (2) remove the within-group correlation for STs. I would advocate the latter for simplicity and because there were no group differences in corticosterone noted in the analysis provided in Fig. 3A.

2) It would be informative to correlate the change in corticosterone levels (Post - Pre) as a function of the change in the PavCA index. This perhaps is a more meaningful analysis than the one provided in Fig. 3C.

3) Lines 13-14 of the abstract and a similar statement in the discussion (lines 464-466) states ‘...baseline corticosterone increases with Pavlovian learning, and that this increase is positively associated with the development of sign-tracking.’ But this is not supported by current analyses in the study. To examine ‘baseline corticosterone increases’ one must calculate the change in corticosterone levels (See comment #2). The second potential issue with this statement relates to Comment #1. The data should be analyzed independently for the 3 categories (ST/GT/IR) and/or analyzed all together with a single linear regression.

4) The authors might consider examining if the GR levels in the ventral hippocampus are (or are not) related to the change in Pavlovian conditioning, similar to the corticosterone analysis that is performed in the current Fig. 3C.

Stylistic suggestions (feel free to disregard).

1) The paper comes across as being written to NIMH. The scientific rationale in the introduction is heavily focused on addressing the negative valence RDoC criteria, which in my opinion is unnecessary. If anything you highlight a weakness of the criteria when you mention that corticosterone is marker of negative valence according to the NIMH but then highlight corticosterone is involved with a host of other behaviors that are not categorized as negative valence (lines 72-80). As another example, I find it to be more direct and informative to say that you are examining ‘anxiety’ or ‘anxiety-like behavior’ instead of ‘behavioral profiles associated with negative valence.’ Overall, the scientific rationale is already in the manuscript. I would recommend minimizing the focus on criteria outlined by a funding agency.

2) I would suggest avoiding the use of ‘appear to be predictive’ in regard to correlational analyses (lines 376-7; 379). The use of ‘predictive’ leads one to infer a causal relationship, which cannot be determined with a correlational analysis.

Reviewer #2

These studies are not hypothesis driven, they simply characterize corticosterone concentrations under different conditions in goal- and sign-tracking animals. The studies would have been more compelling if corticosterone concentrations were manipulated.

Major Concerns

• This is an entirely descriptive study. It would have benefitted from a mechanistic examination of the role of corticosterone in the phenotypic differences between goal- and sign- trackers.

• Results of correlations between post-conditioning corticosterone concentrations with behaviors is significant regardless of phenotype. The authors state that corticosterone levels are higher in sign tracking animals. However, correlations are significant in both phenotypes so a statement about greater correlation in one phenotype or another cannot be made. Are there differences in slopes?

• The results of the open field experiment are difficult to interpret since animals are spending so little time in center zone compared to the outer zone. It seems that the conditions of the test are extremely aversive for the animals.

• The authors conclude that "...GR function, and presumably that within the ventral hippocampus, appears to play an important role in incentive motivational processes". However, other brain regions were not examined so it is not possible to conclude that GRs in the ventral hippocampus have any role at all in motivational processes. This study would have benefitted from a specific test of this idea of the role of GRs particularly since corticosterone concentrations did not differ between groups.

Minor Concerns

• Unclear why the word stress is often placed in quotation marks. What is that meant to signify?

• Description is required of the food deprivation in the paradigm, if indeed rats were food deprived.

• The MP Biomedicals corticosterone kit, if followed verbatim, is not sensitive to low levels of corticosterone. For example, the low value on the curve is stated to be 25ng/ml which could still over-estimate some very low concentrations. Lower concentrations could also be read as below the sensitivity of the assay.

• Vocalization to tail nick in and of itself does not mean the sample should be discarded. Any increases in corticosterone cause by the stress or adverse reaction to nail nick would not be represented in the sample being collected.

• The ventral hippocampus should be shown in Figure 1B.
